# The Sunday Fund Grants and Hospital Expenditure

**Published:** 1905-08-19

**Authors:** 


### The Sunday Fund Grants and Hospital Expenditure.

The awards of the Hospital Sunday Fund this year have caused some comment in hospital circles on the ground that the difference in the amount of grants given to certain hospitals nearly identical in circumstance and size is remarkable, and carries no explanation or justification on the face of the figures published by the Fund. It is further felt that the publication of the approximate cost per head of in- and out-patients, as stated by the hospital authorities, without any notes or comments as to the differences in system which prevail, is very misleading and in some cases unfair to the best managed hospitals. It may be well therefore to deal briefly with both points, and to ask that an explanation may be forthcoming in due course from those immediately concerned.

Dealing first with the differences in the amount of the grants, it will be observed that although the number of beds in the Royal Free and Charing Cross Hospitals are almost identical, and the same may be said of the Great Northern Central Hospital, there is a difference of some £180 in the amount of the awards made as between the highest and lowest grants to these three institutions. Again, how is it that St. George's Hospital, with 351 beds, receives a grant of less than £2,300, when St. Mary's Hospital, with 281 beds, has a grant of nearly £3,000? Or take again the case of the Seamen's Hospital, to which is attached the School for Tropical Diseases, which should give this institution a special claim in Empire days like the present. The Seamen's Hospital with 269 beds only gets £1,745, whereas the Middlesex Hospital with 296 beds, that is only 27 more, receives no less than £3,235. It is a curious fact too that a special hospital like the Chest Hospital, Victoria Park, containing the same number of beds as Charing Cross and the Royal Free, should receive a much smaller grant than either of these institutions. There are other and striking differences which are not explicable to those who have the widest knowledge of the inner working of the institutions immediately concerned, but we have given sufficient instances to indicate the direction in which a detailed explanation of the figures is desirable. No general statement of the basis of award will afford the institutions or their supporters the precise information without which it is impossible for them to understand the grants or their meaning *vis-à-vis* with efficiency.

Again, it is absolutely essential, so long as certain hospitals like the London and the Middlesex persist in refusing to supply certain necessary articles of food to their patients, that any statistics as to the cost per patient or per bed issued by the Central Funds should distinctly indicate this omission, as the practice represents a saving in cost of something like £2 per bed per annum. No one can deny that tea, sugar and butter in these days are essential dietary requisites for all hospital patients, and any hospital committee which tries to save some £2,000 a year of necessary expenditure by depriving its patients of these essential articles of food at the hospital's expense clearly fails to do its duty. The expenditure thus saved represents at least £2 per bed, and should be added to the cost of every hospital where this obsolete and nasty system is continued. It is no answer to urge that the Samaritan Fund provides tea, butter and sugar for those patients who are unable to afford it themselves. Such a contention merely gives the whole case away. Every hospital which really confines its relief to poor people who cannot afford to obtain it elsewhere must necessarily give all the in-patients tea, sugar and butter as part of their ordinary diet. We heard of a complaint of one such hospital this week, where the tea was made in a large urn for the whole of the patients of a ward. A nurse went round to each locker and took out a teaspoonful of the tea provided by each patient, and the whole of this extraordinary sample of so-called tea was then put into the urn, water was added, and the product represented the hospital tea supplied to the patients. It is quite unintelligible how any person of ordinary intelligence can quietly sit on a committee which permits a state of things like this to go on in the present day.

